# The DAMP Awakens: The Role of Heme and Heme Oxygenase-1 in Organ Transplantation

**DOI:** 10.3390/biom16101495

**Published:** 2026-10-14

**Authors:** Tristan Wagner, Michael N. Thomas, Leo E. Otterbein

**Affiliations:** 1 Department of Surgery, Harvard Medical School, Beth Israel Deaconess Center, 3 Blackfan Circle, Boston, MA 02215, USA; 2 Department of General, Visceral, Thoracic and Transplant Surgery, University of Cologne, Kerpenerstr. 62, 50937 Cologne, Germany; michael.thomas@uk-koeln.de

**Keywords:** carbon monoxide, heme, heme oxygenase-1, ischemia–reperfusion injury, organ transplantation, xenotransplantation

## Abstract

Ischemia-reperfusion injury (IRI) determines early and delayed graft function and long-term allograft survival in solid organ transplantation and is one determinant of outcome that remains modifiable in the operating room. Cell-free heme, a Danger-Associated Molecular Pattern (DAMP), is liberated from hemoproteins as a result of IRI, and overwhelms the haptoglobin, hemopexin scavenging systems. As such, it then intercalates into cell membranes, drives Fenton chemistry, and acts as a DAMP that engages TLR4/MD2, precipitating cell death. Heme also triggers its own clearance through NRF2 and BACH1, which regulate expression of heme oxygenase-1 (HO-1, *Hmox1*), that degrades heme into the bioactive molecules, carbon monoxide (CO), biliverdin and ferrous iron. CO modulates inflammation, inhibits cell death pathways and preserves the microcirculation. Biliverdin and bilirubin act as potent antioxidants, and ferritin sequesters labile, highly reactive iron. The role of heme and HO-1 has been well-studied in models of kidney transplantation where HO-1 induction and, independently, exogenous CO treatment have been shown to attenuate transplant-induced IRI and chronic rejection. Importantly, human HO-1 is a critical component of the multi-transgenic donor pig organs that are being tested in ongoing clinical trials. This review discusses the current state of HO-1 in transplantation, including mechanisms of action, preclinical modeling, and clinical applications.

## 1. Introduction

**Figure 1:**
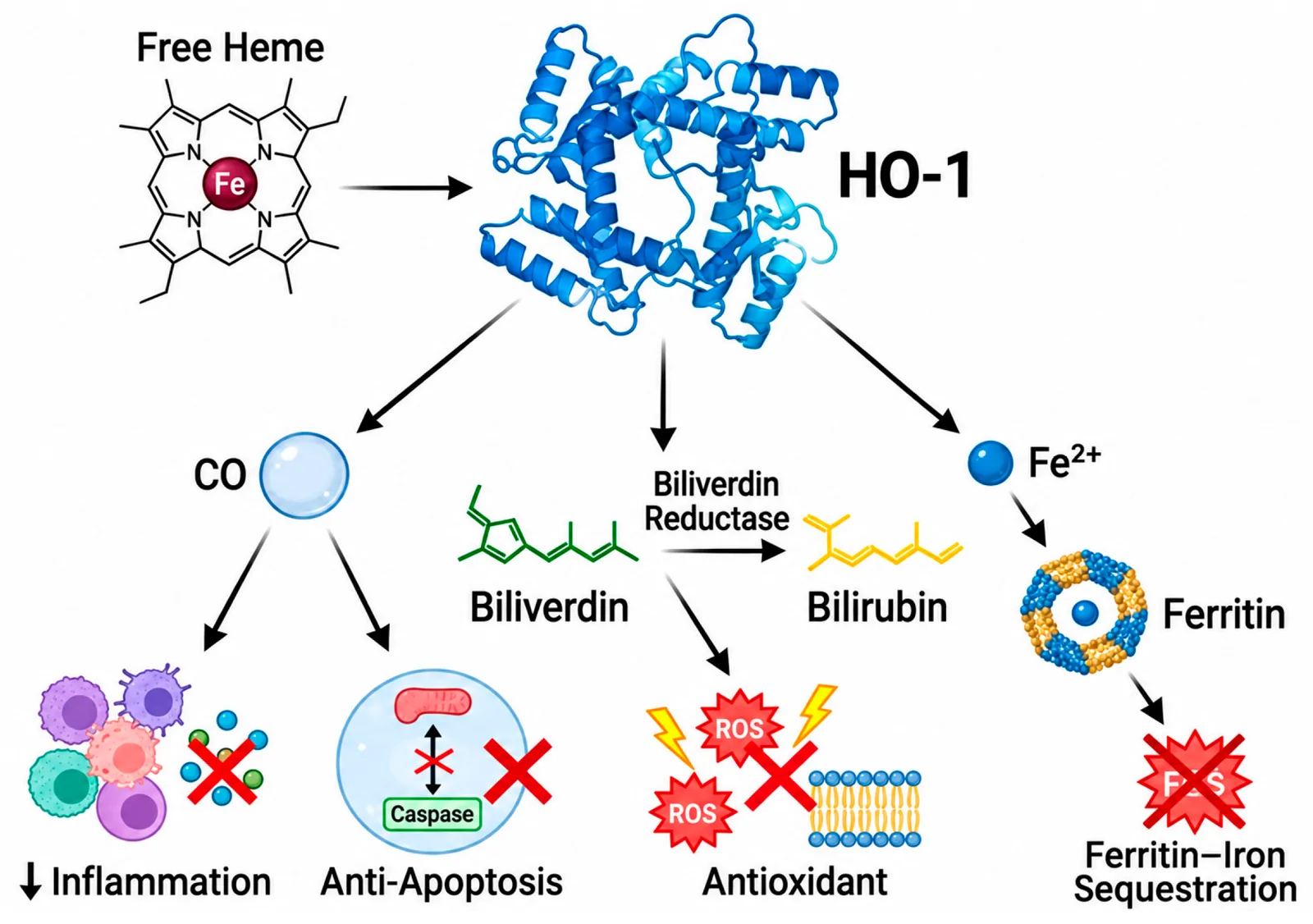
HO-1 catalyzes the rate-limiting step in heme degradation, generating CO, biliverdin (later converted to bilirubin) and Fe^2+^, which drives ferritin synthesis. These metabolites collectively promote cytoprotective pathways, including anti-inflammatory signaling, reduced apoptosis, and attenuation of oxidative stress through iron sequestration and decreased ROS. In the context of organ injury and transplantation, adequate HO-1 activity supports graft protection and lowers acute and chronic rejection, whereas insufficient induction or excessive heme accumulation leads to amplified inflammation, increased cell damage and death, and worsened tissue injury [6]. Created in https://BioRender.com.

Ischemia-reperfusion injury (IRI) remains an enormous challenge in organ transplantation, determining both immediate post-transplant outcomes and long-term allograft and patient survival. Unlike most perioperative insults, IRI is inseparable from the operation itself: it begins with cross-clamping in the donor, sustained through cold storage and back-table preparation, and is then exacerbated at the moment of reperfusion where hyperemic events create a pro-oxidative environment. Its severity is therefore governed by variables that include the quality of the flush, cold and warm ischemia time, duration of the vascular anastomosis, and the choice to accept or decline a marginal graft, which makes it the one component of transplant outcome that is modifiable in the operating room [1,2,3]. Among the endogenous cytoprotective mechanisms that occur during such cellular stress, the heme oxygenase-1 (HO-1) system is a potent defense mechanism that acts in a manner that befits the needs of the cells and tissue to ensure survival and function of the organ [4,5]. HO-1 catalyzes the first step in the degradation of heme, yielding three biologically active products: carbon monoxide (CO), biliverdin-IXα (subsequently reduced to bilirubin-IXα by biliverdin reductase) and ferrous iron (Fe^2+^), which induces ferritin activity resulting in chelation of the heme iron (Figure 1) [6,7].

Each enzymatic product of HO-1 has cytoprotective functions that include antioxidant defense, anti-inflammatory signaling and anti-cell death mechanisms, while preserving microcirculatory integrity [4,8]. The importance of the HO-1 system is its unique ability to prolong transplant survival and its enzymatic products, particularly CO, which suppresses rejection and promotes immune tolerance [9]. It is evident that if HO-1 is absent, tolerance cannot be achieved despite optimal immunosuppression [5,9,10,11]. This review focuses on the HO-1/heme axis in the setting of organ transplantation, with up-to-date experimental evidence across multiple organ systems that includes allotransplantation, xenotransplantation, and emerging therapeutic strategies that seek to leverage the power of this gene and its potent metabolic products [10]. From a surgical perspective: the questions we ask throughout are where in the operative and preservation sequence the HO-1 pathway can be leveraged. We first outline the two phases of IRI, the cell death programs they engage and the role of cell-free heme as the central DAMP of this process. We detail the structure, regulation and cytoprotective mechanisms of HO-1, survey the organ-specific evidence in allotransplantation and xenotransplantation and we conclude with the translational strategies including gene therapy and CO delivery modalities, that are most likely to convert this biology into clinical benefit.

## 2. Ischemia-Reperfusion Injury and Heme as a DAMP

### 2.1. The Ischemic and Reperfusion Phases and Regulated Cell Death

Ischemia-reperfusion injury has two phases: the ischemic phase, where blood flow is stopped and the tissue becomes rapidly hypoxic, followed by the reperfusion phase, where blood flow is restored, leading to hyperemia and a sudden hyperoxic state. Each phase contributes distinct but interrelated mechanisms of cellular damage, and together these generate the oxidative and inflammatory milieu that determine graft survival both short and long-term [3]. Tissue damage in this setting promotes the release of damage-associated molecular patterns (DAMPs), endogenous signals that alert and activate the innate immune system, thereby amplifying sterile inflammation. Among the various DAMPs implicated in IRI, including mitochondrial components and nuclear proteins, heme has emerged as a central bioactive mediator and is the focus of this review [12]. Cessation of blood flow at procurement deprives the tissue of oxygen and nutrients, triggering rapid depletion of adenosine triphosphate (ATP) and exhaustion of aerobic respiration. The resulting transition to anaerobic glycolysis increases lactate levels and intracellular acidosis, disrupting pH-dependent enzymatic functions. ATP depletion impairs energy-dependent ion pumps, particularly the Na^+^/K^+^-ATPase and Ca^2+^-ATPase, resulting in loss of critical ionic gradients. The sudden influx of sodium and calcium activates deleterious enzymatic cascades, including phospholipases, proteases and endonucleases that initiate programmed cell death [13,14,15]. In parallel, loss of oxidative phosphorylation leads to accumulation of succinate driven by a metabolic reversal of mitochondrial complex II and the TCA cycle, priming mitochondria for a subsequent oxidative burst through reversal of electron transport at complex I [16,17]. Mitochondrial swelling culminates in mitochondrial permeability transition pore (mPTP) formation, which dissipates the membrane potential and releases cytochrome c into the cytosol [18,19]. This is the biochemical correlate of what surgical practice records simply as cold ischemia time, and it explains why each additional hour of storage translates into measurable graft dysfunction [20]. Restoration of blood flow precipitates an even more destructive phase through explosive generation of reactive oxygen species (ROS) and DAMPs, driven by the sudden reintroduction of molecular oxygen [21]. The burst of oxygen to ischemia-damaged mitochondria generates massive quantities of superoxide anion (O_2_^−^) through electron leakage [22,23]. Superoxide dismutase converts O_2_^−^ to hydrogen peroxide, which, in the presence of iron, generates highly reactive hydroxyl radicals capable of indiscriminate oxidative damage to lipids and proteins [24]. Oxidative stress simultaneously activates two opposing transcriptional programs: nuclear factor-κB (NF-κB), which initiates transcription of pro-inflammatory genes [25] and nuclear factor erythroid 2-related factor 2 (NRF2), the master regulator of antioxidant defense that induces cytoprotective genes including HO-1 [26,27]. ROS-mediated activation of the NLRP3 inflammasome triggers caspase-1-dependent maturation of interleukin-1β and IL-18, promoting pyroptosis [28,29], while iron released from heme-containing proteins catalyzes the lipid peroxidation that executes ferroptosis [30,31]. Endothelial cells upregulate ICAM-1, VCAM-1 and E-selectin, triggering MyD88-dependent NF-κB activation, leukocyte adhesion and a cytokine storm [32], with recipient monocytes and neutrophils infiltrating the graft within minutes of unclamping. Cell death determines how much of the injury activates the recipient immune response. Apoptosis proceeds through intrinsic and extrinsic mechanisms. mPTP opening releases cytochromes c and procaspase-9 that form the apoptosome, while Fas (CD95) and TNF receptor 1 activate caspase-8 and are immunologically silent, although it can transition into secondary necrosis in the presence of overwhelming stress. Necroptosis occurs when caspase-8 is inhibited. Receptor interacting protein kinases (RIPK) 1 and 3 form the necrosome, phosphorylating mixed lineage kinase domain-like protein (MLKL), which, in turn forms plasma membrane pores [33]. Unlike apoptosis, necroptosis releases DAMPs including HMGB1, ATP, heme and mitochondrial DNA, provoking a far more severe inflammatory response that begins during ischemia as cells begin to die from lack of oxygen [34,35]. Mitochondrial DAMPs are particularly potent because their bacterial ancestry allows them to engage the same receptors such as TLR4 [36]. Pyroptosis follows inflammasome activation and gasdermin pore formation and the recently described PANoptosis integrates elements of pyroptosis, apoptosis and necroptosis in a single coordinated program [37,38]. Ferroptosis, driven by excess free iron and impaired glutathione peroxidase-4-mediated defense, is likely elicited by the presence of free heme and is increasingly regarded as a therapeutic target in graft injury [39,40]. Ultimately, the balance between lytic cell death and more controlled, non-inflammatory pathways determines whether tissue injury progresses or resolves within the first hours after transplantation is complete [22,34,41].

### 2.2. Heme Pool and Scavenging Systems

While protein-bound heme is essential for oxygen transport, electron transfer and enzymatic catalysis and additionally serves important roles in signal transduction and functional regulation, cell-free heme becomes profoundly toxic when released from its intracellular protein-bound components [42]. Heme also functions as a sensory cofactor in heme-based gas-sensing proteins, including the bacterial oxygen sensors FixL, EcDOS, and HemAT. In these proteins, binding of O_2_ and other gaseous ligands to the heme iron induces conformational changes that regulate downstream signaling pathways [43]. During IRI, mechanical trauma to erythrocytes, disruption of cellular hemoproteins and lysis of parenchymal cells result in substantial accumulation of labile heme in the extracellular milieu [44]. The lipophilic nature of heme enables intercalation into cellular membranes, disrupting membrane integrity and promoting lipid peroxidation [45]. Its central iron atom, oscillating between ferrous and ferric states, generates hydroxyl radicals that damage all cellular macromolecules. Beyond direct cytotoxicity, free heme functions as a prototypical DAMP, activating innate immune responses through pattern recognition receptors such as TLR4 [46,47,48].

The quantitative contribution of individual hemoproteins to the labile heme pool differs by orders of magnitude and the compartment in which each protein resides determines both the kinetics and the anatomical site of heme release during reperfusion (Table 1).

Hemoglobin is by far the dominant reservoirs for heme. Erythrocytes trapped within the graft microcirculation, together with residual blood remaining after the organ flush, constitute a principal source and is a direct link between procurement technique and the heme burden the graft must later handle. Myoglobin, as a second major source of heme, becomes relevant in cardiac and skeletal muscle injury. Within the cell, the cytochrome P450 system also represents a large heme sink, while mitochondrial cytochromes are released as mitochondria undergo permeability transition and autophagic turnover, precisely in line with the events that characterize the ischemic phase. Several hemoproteins function as regulatory heme sensors rather than quantitative reservoirs. BACH1 links heme abundance directly to HO-1 transcription while and NPAS2, mPER2 and Rev-ErbB regulate circadian rhythm [44,45].

Heme Regulated Inhibitor or HRI is an intracellular heme sensor that, when heme is scarce, phosphorylates the α-subunit of eukaryotic initiation factor 2 and thereby halts globin translation, preventing the accumulation of heme-free globin chains that would otherwise precipitate and cause proteotoxicity [56]. HRI was the first member of the eIF2α kinase family, and its activation can silence globin synthesis. Beyond heme deficiency, HRI is also activated by oxidative stress and by denatured proteins, both of which are prominent features of ischemia–reperfusion stress [56]. Tryptophan catabolism in immune/inflammatory contexts; heme binding is required for formation of the catalytically active IDO1 holoenzyme [60]. This pathway operates independently of heme deficiency, thereby linking mitochondrial injury directly to the integrated stress response [49]. HRI-mediated stress signaling has also been demonstrated in non-erythroid cells, including epithelial cells, suggesting that its function extends beyond the regulation of globin synthesis to the maintenance of cellular proteostasis during mitochondrial stress [56]. In the setting of ischemia–reperfusion, HRI may therefore represent a mechanistic link between heme dysregulation, oxidative and mitochondrial stress, and the cellular adaptive response, although the specific contribution of the HRI and the stress response in renal IRI remains to be defined.

**Figure 2:**
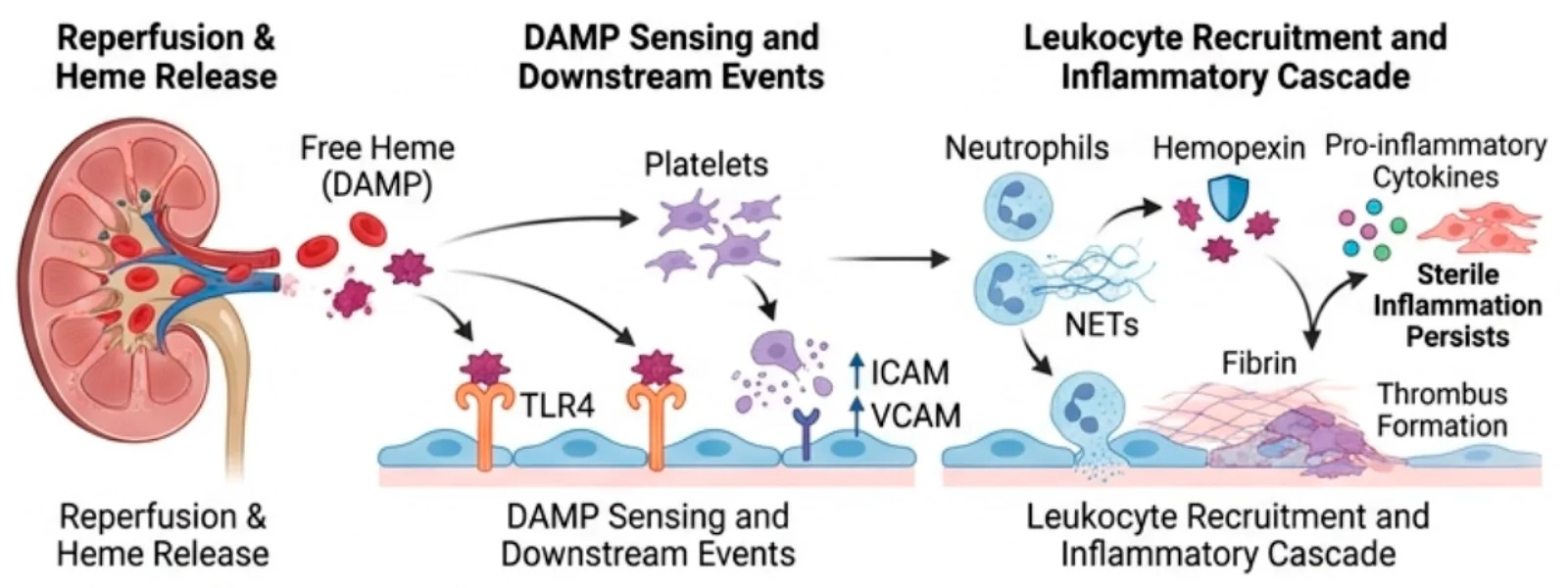
Heme as a damage-associated molecular pattern (DAMP) in kidney transplantation. Reperfusion following kidney transplantation triggers the release of cell-free heme into the extracellular space, where it acts as a potent DAMP. Heme activates TLR4 on endothelial cells and platelets, driving neutrophil recruitment and the formation of neutrophil extracellular traps. Activated endothelium upregulates adhesion molecules and promotes platelet aggregation, fibrin deposition and thrombus formation. Hemopexin partially scavenges circulating free heme, yet the inflammatory cascade persists, culminating in sterile inflammation, cytokine release, and activation of mesangial cells within the glomerulus. Created in https://BioRender.com.

The host response to free heme operates through a hierarchical, multi-layered scavenging system in which distinct plasma proteins intercept hemoproteins and free heme at defined points. The first-line defense against extracellular hemoglobin is haptoglobin, an abundant plasma glycoprotein synthesized primarily by hepatocytes [61]. Haptoglobin binds hemoglobin and is one of the strongest known non-covalent protein-protein interactions, forming a complex too large for glomerular filtration. Haptoglobin stabilizes the bound hemoglobin dimers and enforces a closed heme pocket, preventing heme release even when the iron is oxidized. The complex is cleared through CD163, a scavenger receptor confined to the monocyte-macrophage lineage and most highly expressed on Kupffer cells, splenic red pulp macrophages, resident bone marrow macrophages and alveolar macrophages. Within the macrophage lysosome, liberated heme is processed by HO-1, generating CO, biliverdin and ferrous iron (Figure 2) [62].

When haptoglobin capacity is exhausted, heme dissociating from oxidized methemoglobin and other hemoproteins accumulates extracellularly, where it is then captured by hemopexin, which is the plasma protein with the highest known affinity for heme. Hemopexin prevents heme and iron from driving oxidative reactions, engaging TLR4 on endothelium and leukocytes, and from partitioning into lipophilic membranes (Figure 2) [48,63,64,65,66].

The hemopexin-heme complex is internalized by hepatocytes via CD91-mediated endocytosis and catabolized by HO-1, and hemopexin recycles after delivery, permitting repeated rounds of scavenging. Beyond the liver, hemopexin is active in skeletal muscle, the nervous system, the retina and the kidney, contributing local sequestration at the sites most vulnerable to IRI [67,68]. Secondary scavengers take over once primary capacity is exhausted. Alpha-1-microglobulin binds heme, scavenges ROS and is internalized by proximal tubular cells, where it localizes to mitochondria and protects respiratory function against heme-driven oxidative damage while transferrin and ferritin sequester the iron within the graft itself. In transplantation this hierarchy is routinely overwhelmed, since procurement, cold storage, cardiopulmonary bypass and reperfusion of a large vascular bed consume scavenging capacity faster than it can be replenished by the liver [48].

The TLR4-heme axis links cellular damage directly to innate immune cell activation in IRI [48]. TLR4, classically kown for lipopolysaccharide recognition, also binds heme through its leucine-rich repeat domains, with myeloid differentiation factor 2 (MD-2) as co-receptor [69]. Upon heme binding, TLR4 dimerizes and recruits MyD88 and TRIF, initiating parallel cascades. The MyD88-dependent pathway activates IL-1 receptor-associated kinases and TNF receptor-associated factor 6, leading to IκB kinase activation and NF-κB nuclear translocation [70]. Nuclear NF-κB induces TNF, IL-1β, IL-6, cyclooxygenase-2, inducible nitric oxide synthase and adhesion molecules that produce an innate immune response that manifests clinically as early graft dysfunction. TLR4 deficiency or antagonism significantly ameliorates IRI across organ systems [71].

## 3. The Heme Oxygenase-1 System

### 3.1. Enzymatic Mechanism and Transcriptional Regulation

HO-1, encoded by *HMOX1*, is a 32-kDa enzyme. Unlike the constitutively expressed isoform HO-2/*HMOX2*, found primarily in brain and testis, HO-1 is highly inducible by diverse stress stimuli [72]. The enzymatic reaction proceeds through three sequential oxidation steps, each consuming one molecule of O_2_ and requiring electrons from NADPH-cytochrome P450 reductase, and generates equimolar amounts of biliverdin-IXα, carbon monoxide and iron [73]. Biliverdin is rapidly converted to bilirubin via biliverdin reductase (Figure 1).

Importantly, it remains unclear whether HO-1 expression always reflects enzymatic product formation, which depends on the availability of labile heme as a substrate. Accordingly, increased HO-1 expression indicates enhanced capacity for heme degradation rather than necessarily increased enzymatic flux [72,73]. Quantitative in vivo data on labile heme and the corresponding generation of CO and biliverdin during transplantation remain limited [44,63].

The inducibility that distinguishes HO-1 from HO-2 reflects an unusually complex, multi-element stress-responsive promoter, and its architecture explains why the enzyme responds to precisely those signals that define IRI [74]. Under basal conditions, the enhancer regions of HO-1 are occupied by the transcriptional repressor BACH1 (Table 1). Heme binds BACH1 directly, releasing it from DNA and permitting occupancy by NRF2, which is itself stabilized when oxidative and electrophilic stress modifies its cytosolic partner KEAP1 [26]. The result is a dual-switch system in which the two proximate hallmarks of reperfusion, free heme and ROS, converge on the same promoter and derepress and activate transcription simultaneously. Additional inputs from NF-κB, hypoxia signaling and MAPK cascades act on these same regions in a stimulus- and cell-type-dependent fashion [72,75].

### 3.2. Cellular Distribution of HO-1 Induction

HO-1 is a central endogenous cytoprotective molecule induced in response to IRI, and genetic loss-of-function models substantiate this, where HO-1-deficient animals display markedly increased susceptibility to IRI, whereas restoration or overexpression confers protection across organ systems [76]. Its induction is driven by the very signals that define IRI, free heme released as a DAMP, NRF2 activation and pro-inflammatory cytokines [4,77]. In the parenchyma, the metabolically and most exposed cells respond first including renal proximal tubular epithelium, hepatocytes and cholangiocytes as well as cardiomyocytes, in each case with reduced apoptosis, preserved mitochondrial function and improved early function [39,77,78,79,80,81]. Endothelial HO-1 suppresses ICAM-1 and VCAM-1 and limits leukocyte transmigration, while smooth muscle HO-1 modulates vascular tone and inhibits hyperproliferation that underlies chronic vascular remodeling that develops into transplant vasculopathy [5,82]. HO-1 expression in macrophages shifts the phenotype from an M1-like to an M2-like and restrains dendritic cell maturation and migration, thereby limiting the priming of donor-reactive T cells. Of note, myeloid-restricted expression alone is sufficient to modulate the severity of renal IRI [83,84,85]. These differential effects of HO-1 across cell types make it unusual among cytoprotective genes where a single pathway simultaneously limits parenchymal cell death, preserves the microvasculature and dampens the transition from innate injury to adaptive alloimmunity [5,11,45].

### 3.3. Cytoprotective Mechanisms in Transplantation

**Figure 3:**
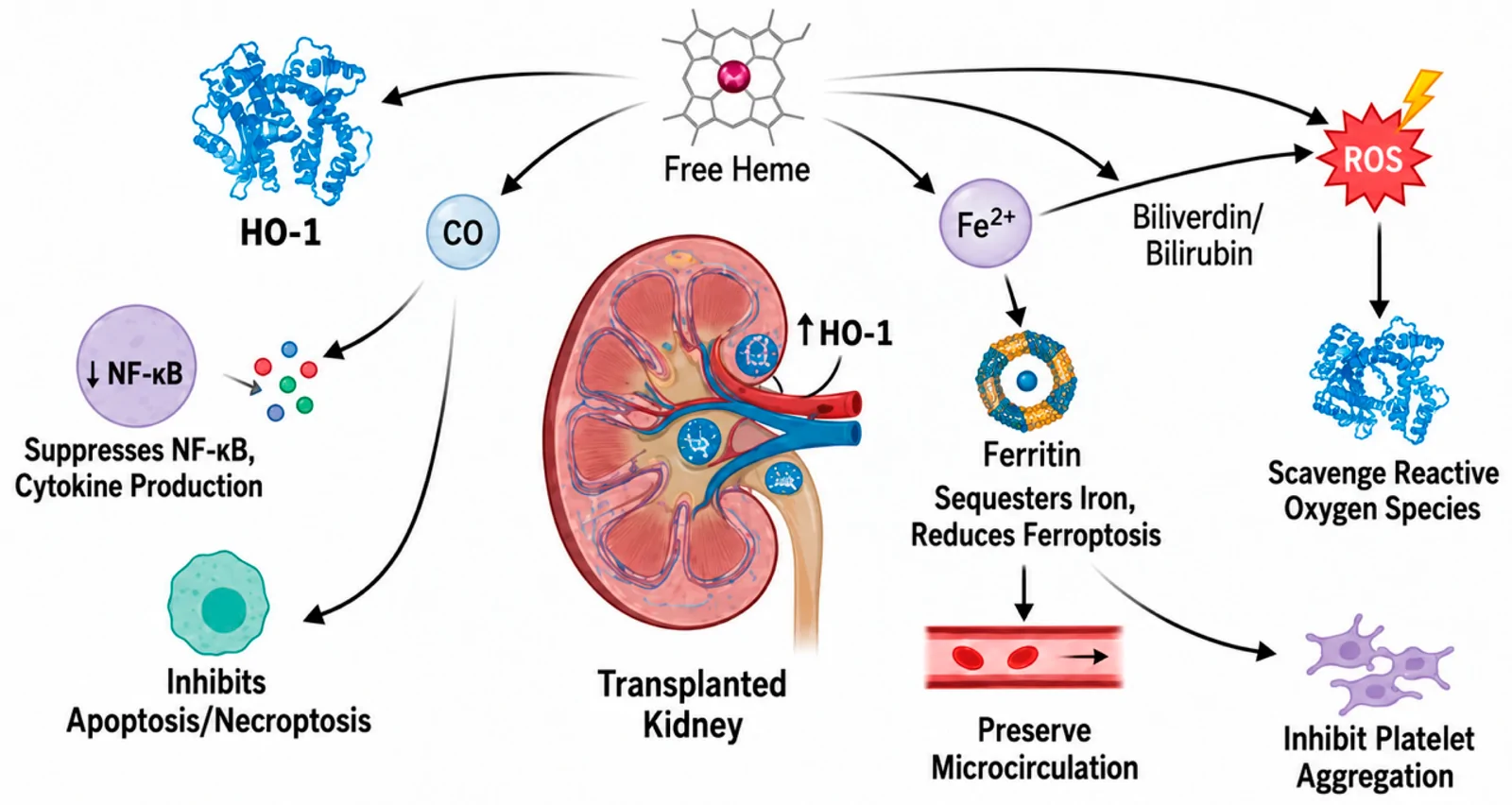
Cytoprotective mechanisms of the HO-1 system in the transplanted organ. Degradation of free heme by HO-1 removes a pro-inflammatory DAMP and yields CO, biliverdin/bilirubin and ferrous iron, which act in concert to suppress cytokine production and NF-κB signaling, inhibit apoptotic, necroptotic and ferroptotic cell death, scavenge reactive oxygen species, sequester labile iron through ferritin induction, and preserve microcirculatory perfusion through vasodilation and inhibition of platelet aggregation. Created in https://BioRender.com.

HO-1 exerts anti-inflammatory effects through multiple pathways (Figure 3). Degradation of heme eliminates a potent pro-inflammatory DAMP, reducing TLR4-mediated signaling, while CO acts through MAPK pathways, particularly p38 MAPK [82,83,84,85,86].

The cytoprotective effects of HO-1 are product- and context-dependent. Exogenous CO exerts cytoprotective effects [80,87]. Similarly, the bile pigments biliverdin and bilirubin exert antioxidant and anti-inflammatory effects. Exogenous biliverdin has been shown to attenuate IRI in experimental transplantation models, with protective effects observed independently of HO-1 induction. These findings suggest that bile pigments can reproduce at least part of the cytoprotective effects associated with HO-1 activity [88,89]. Beyond its effects on canonical signaling pathways, CO can also directly interact with heme-containing sensor proteins and thereby modulate their function. CO inhibits TNF, IL-1β and IL-6 production while promoting IL-10 expression. Cystathionine β-synthase (CBS), which contains a regulatory heme cofactor, is inhibited upon CO binding, while other heme-based CO-responsive proteins include CooA, RcoM, and NPAS2, illustrating how heme can translate CO binding into functional cellular responses [43,90]. Biliverdin/bilirubin act as antioxidants [44,91] while Fe^2+^ released as the third product of heme catabolism is rapidly sequestration by ferritin to prevent pro-oxidant injury [5,72,92].

HO-1-driven M2 polarization further upregulates IL-10 and TGF-β, downregulates iNOS-derived nitric oxide, and enhances the clearance of apoptotic debris that prevents secondary necrosis and limits further DAMP release into the graft [79,87]. Parenchymal cell death is one of the causes of early graft dysfunction. HO-1/CO inhibit apoptosis in part by activating p38 MAPK and upregulating Bcl-2 and Bcl-xL while suppressing Bax and caspase-3 [93]. This effect is seen in hepatocytes, renal tubular cells, cardiomyocytes and islet beta-cells, indicating a common mechanism conserved across these cell types [94,95]. HO-1 induction in the kidney attenuates necroptotic signaling by suppressing RIPK3 and reducing MLKL membrane translocation, limiting DAMP release that would otherwise amplify this cascade [39]. Ferroptosis is antagonized through two complementary routes that include free iron liberated during heme catabolism, which is subsequently sequestered by ferritin, removing the substrate for Fenton-driven lipid peroxidation. The bile pigments, biliverdin and bilirubin, act as a regenerating redox couple in which bilirubin scavenges lipid peroxyl radicals and is restored, albeit controversially, from biliverdin by biliverdin-reductase, allowing one molecule to detoxify multiple radicals [44,91]. The same dependency defines the limits of the system. When ferritin induction lags behind the rate of heme catabolism, the labile iron pool expands and HO-1 transitions to become pro-oxidant rather than protective [5,72,92].

Preservation of the microcirculation is equally decisive, since a technically perfect anastomosis does not guarantee capillary perfusion. CO activates soluble guanylate cyclase (cGMP) in vascular smooth muscle, increasing cyclic GMP driving vasodilation, inhibiting platelet activation and aggregation thus reducing microthrombosis during the critical post-reperfusion period [77,80,87]. CO also prevents transplant-associated vascular stenosis, a major cause of late graft loss. In a rat aortic transplant model, low-dose CO inhibited intimal hyperplasia by over 60% with marked suppression of leukocyte, CD68^+^ macrophage and CD3^+^ T cell infiltration, and a single one-hour pre-exposure suppressed intimal hyperplasia after carotid balloon angioplasty by approximately 74%. Conversely, *Hmox1^−/−^* mice developed occlusive thrombi significantly faster than wild-type controls after vascular injury, with elevated tissue factor, von Willebrand factor, increased endothelial apoptosis and greater ROS accumulation. Transplantation of HO-1-deficient bone marrow into wild-type recipients reproduced the prothrombotic phenotype [96].

## 4. Allotransplantation and Xenotransplantation

The HO-1/CO axis is one of the few endogenous stress-response systems with demonstrated relevance across all major transplantable organs, making it a coherent target for donor conditioning, machine perfusion supplementation and recipient-side therapy. How strongly HO-1 can be induced varies between individuals, because two common promoter variants set its activity: a (GT)n repeat (rs3074372), in which fewer repeats give a stronger response to oxidative stress, and the A(-413)T single nucleotide polymorphism (rs2071746), in which the A allele raises promoter activity [97,98]. In transplantation the effect lies with the donor genotype rather than the recipient’s, which is what would be expected if the graft has to process the heme it releases. Recipients of a kidney from a short-allele donor had lower serum creatinine at one year [99] and better graft function up to three years [100] and their grafts survived longer with fewer losses to chronic allograft nephropathy after long cold ischemia [101]. The same is true in the liver, where among 308 transplants donor A-allele carriage was associated with one-year graft survival of 84% versus 63% for TT donors, fewer losses to primary dysfunction and higher hepatic HO-1 mRNA [102]. The donor repeat pattern therefore points back to the graft itself, whose organ-specific behaviour is considered next.

Heme oxygenase-1 (HO-1) has emerged as one of the most extensively studied cytoprotective systems in experimental and clinical organ transplantation. By catabolizing free heme into biliverdin, carbon monoxide (CO), and ferrous iron, HO-1 and its downstream products confer broad protection against ischemia-reperfusion injury, the dominant driver of early graft dysfunction across kidney, liver, heart, small bowel, skin, lung and even islet cell transplantation. Decades of work in rodent and large-animal models have shown that HO-1 induction, whether through genetic overexpression, pharmacologic inducers such as cobalt protoporphyrin, or gene therapy, consistently improves graft survival, reduces apoptosis and necrosis in donor tissue, and dampens the innate immune response triggered by cell death and oxidative stress. Its enzymatic products contribute independently to these benefits. HO-1 upregulation also modulates adaptive immunity, favoring regulatory T cell expansion and dampening dendritic cell maturation, which together attenuate alloimmune responses and promote long-term graft acceptance. This body of evidence, summarized in Table 2, and condensed below details the translational strategies studied including ex vivo machine perfusion supplemented with CO or biliverdin, pharmacologic HO-1 induction in donors before organ procurement, and CO-releasing molecules as adjunct therapies, positioning the HO-1 system as a promising target for reducing IRI and improving both short- and long-term outcomes.

### 4.1. Kidney Transplantation

The kidney is particularly vulnerable to IRI because of its high metabolic demand, complex microvascular architecture and the marked oxygen gradient across the renal cortex and medulla. Delayed graft function remains the dominant early complication of deceased-donor transplantation and an established risk factor for acute rejection and inferior long-term survival [2,123].

Induction of HO-1 is protective in rat renal allografts where it attenuates IRI-induced apoptosis and improves survival [77] and protection extends to optimal grafts as with young donors as well as marginal grafts from aged animals which are experimentally analogous to an extended-criteria donor and thus HO-1/CO is attractive for expanding the usable donor pool. The benefit is not confined to the perioperative window. HO-1 induction also reduces the structural changes of chronic rejection, including interstitial fibrosis, tubular atrophy and arteriosclerotic lesions, with reduced graft infiltration by monocytes/macrophages and CD8^+^ T cells, decreased TNF and increased anti-apoptotic factor expression [39].

In rat kidney transplantation, CO inhalation protected grafts preserved in cold University of Wisconsin solution for 24 h, preventing functional deterioration and supporting recipient survival, with reduced oxidative stress, decreased apoptosis, and improved microcirculatory function [103]. Low-dose CO also inhibits progressive chronic allograft nephropathy and restores renal allograft function in established disease, extending the therapeutic window beyond preconditioning to the treatment of chronic dysfunction [104]. In swine, intraoperative administration of inhaled CO reduced delayed graft function in pig kidney allografts [105] and ex vivo application of CO in UW solution prevented transplant-induced renal IRI [124] critically demonstrating that conditioning of the graft alone, with no donor or recipient exposure, is sufficient for long-term survival. CO-releasing molecules have been shown to deliver comparable benefits, improving glomerular filtration rate and tubular sodium and glucose reabsorption in cold-stored kidneys [125]. The broader case for the use of gas transmitters in renal transplantation, encompassing CO alongside nitric oxide and hydrogen sulfide, remains an active area of study [126].

### 4.2. Liver Transplantation

Overexpression of HO-1 improves liver function and graft survival after prolonged cold preservation by attenuating IRI [78] and adenoviral gene transfer protected steatotic rat livers [107]. More recent work focused on the role of the macrophage, where myeloid cell-derived HO-1 controls polarization toward an anti-inflammatory phenotype, attenuating hepatic inflammation and pyroptosis in ischemia-stressed livers, and adoptive transfer HO-1-modified bone marrow-derived macrophages prevents hepatic IRI [79,108].

### 4.3. Thoracic Organ Transplantation: Heart and Lung

Heart and lung transplantation both share similar challenges with regard to transplantation. They tolerate ischemia poorly and in both, early graft dysfunction is the leading cause of neointimal formation and graft stenosis caused by vascular smooth muscle proliferation and leukocyte infiltration [80]. Adenoviral- and adeno-associated viral-mediated HO-1 transfer prolonged cardiac allograft survival and inhibited graft arteriosclerosis, with protection correlating with NF-κB downregulation and reduced apoptosis [81,109,127].

In the lung, HO-1 protects the alveolar-capillary interface from IRI and from primary graft dysfunction [128]. Induction of HO-1 or administration of inhaled CO preserves pulmonary architecture by reducing alveolar edema, fibrin deposition and inflammatory infiltration, and markedly attenuates hyperoxic lung injury, preventing pleural effusion, reducing protein leakage into the airway, and preserving histological architecture [113]. Mechanistically, CO interrupts MAPK-driven expression of early growth response and its downstream targets in ischemic lungs, limits cytokine production and apoptosis, while suppressing neutrophil recruitment into the airways [112,114]. Exogenous CO also mitigates chronic allograft rejection and fibrosis after lung transplantation, addressing the chronic lung allograft dysfunction that limits long-term survival [129].

### 4.4. Islet, Intestinal and Skin Transplantation

Both HO-1 induction and exogenous CO administration have been shown to improve islet survival and graft function through distinct mechanisms, including protection of β-cells from apoptosis via cyclic GMP-dependent signaling [117]. Treatment of donors, isolated islets or recipients prolongs islet allograft survival while suppressing TNF, iNOS and MCP-1 and reducing macrophage infiltration [117]. HO-1-derived bioactive products promote antigen-specific tolerance through regulatory T cell induction and increased Foxp3 and TGF-β expression [119]. CO-releasing molecules modulate immune responses and enhance β-cell survival and regeneration [130]. Islet cell harvest in CO-saturated medium has already been applied in patients undergoing autotransplantation for chronic pancreatitis [118]. In this pilot study, CO treatment resulted in a higher islet oxygen consumption rate, indicating improved islet quality prior to transplantation. CO treatment was associated with reduced early β-cell death following transplantation and favorable changes in inflammatory chemokines. After 6 months, 3 of 10 patients recieving CO-treated islets achieved insulin independence compared with none of the control group, although the small sample size limits definitive conclusions regarding clinical efficacy [118].

Small bowel transplantation remains among the most challenging of organ transplants owing to the heightened immunogenicity of intestinal tissue, its susceptibility to IRI, and the consequences of barrier failure [121,131].

HO-1 induction attenuates intestinal IRI by reducing epithelial apoptosis, oxidative stress and cytokine production [121]. In experimental intestinal IRI, increased HO-1 expression was associated with reduced mucosal injury, oxidative stress and inflammatory cytokine production, supporting a direct cytoprotective role of the HO-1 system during intestinal reperfusion [132]. In this model, HO-1-transduced mesenchymal stem cells significantly reduced acute cellular rejection, apoptosis and NK-cell activity, while suppressing pro-inflammatory and immune-related cytokines and increasing regulatory T-cell numbers [133]. These findings suggest that HO-1 may protect the intestinal graft not only from ischemic injury but also from subsequent alloimmune injury. In addition, HO-1-dependent modulation of dendritic-cell maturation and inflammatory signaling provides a potential mechanistic link between its cytoprotective effects during IRI and its immunoregulatory effects after transplantation [84]. HO-1-transduced mesenchymal stem cells reduced acute rejection and improved outcomes in rat small bowel transplantation [133]. Skin and flap models provide complementary insight into the immunoregulatory functions of HO-1, where induction prolongs allograft and flap survival by suppressing T-cell-mediated responses, inhibiting dendritic cell maturation and reducing local inflammatory signaling [122].

### 4.5. Xenotransplantation

Xenotransplantation has moved from concept to clinical application, with gene-modified pig-to-human kidney and liver transplants recently reported [134,135]. Xenograft rejection presents hyperacute rejection, mediated by preformed antibodies against xenoantigens, acute vascular rejection with endothelial activation and thrombotic microangiopathy and cellular rejection involving T cells and macrophages [136]. Minimizing IRI is now recognized as a distinct requirement in this setting, and HO-1 expression has emerged as a critical component of xenograft protection [137,138]. Early work demonstrated that HO-1 expression within graft endothelial and smooth muscle cells is essential for long-term cardiac xenograft survival [139] and that exogenous CO could rescue xenografts in the absence of HO-1, establishing CO as a key mechanism of protection [9]. Transgenic pigs expressing human HO-1 were subsequently generated and kidneys from these animals resisted rejection during ex vivo perfusion with human blood and transgenic porcine aortic endothelial cells show reduced TNF-mediated apoptosis and decreased ICAM-1, VCAM-1 and E-selectin expression, effects abolished by a selective HO-1 inhibitor [140,141]. Clinical xenotransplantation has incorporated HO-1 into multi-transgenic donors. The pig-to-human cardiac xenotransplant studies used donor pigs expressing human HO-1 alongside xenoantigen knockout, human complement regulators and human thromboregulatory proteins [142]. This donor carried ten genetic modifications in total, including knockout of three xenoantigen genes (GGTA1, CMAH and B4GALNT2) and the growth hormone receptor gene, together with six human transgenes comprising CD46, CD55, thrombomodulin, endothelial protein C receptor, CD47 and HO-1. Critically, the reduction in rejection was not attributable to any single modification but to this unique combination, where xenoantigen knockout removes the substrate for hyperacute rejection yet leaves the endothelium exposed to complement activation, coagulation dysregulation and ischemia-reperfusion injury. It is these residual mechanisms that the cytoprotective and regulatory transgenes, HO-1 among them, together address. Current efforts continue to optimize expression level and tissue specificity [143].

## 5. Therapeutic Translation

### 5.1. Carbon Monoxide and CO-Releasing Molecules

Carbon monoxide, once regarded solely as a toxic gas, is now recognized as a signaling molecule. At low concentrations (10–500 ppm; 0.01–0.5%) it activates specific pathways without toxicity, modulating numerous signaling cascades [144,145,146], which mediate many of its well-described anti-inflammatory and anti-apoptotic effects [115]. Its therapeutic potential has been validated in transplantation where brief exposure to 250 ppm before and after transplantation significantly reduces IRI in kidney, liver and intestinal grafts [104,120] with comparable benefit in thoracic organs [112]. While inhaled CO is effective experimentally, clinical application faces practical challenges of delivery, dosing and monitoring and, not least, the difficulty of proposing what continues to be viewed as a toxic gas, to an anesthesia team. CO-releasing molecules (CORMs) and CO Prodrugs were developed to store and controllably release CO in biological systems [147,148]. Ruthenium-based metal carbonyls have demonstrated protective effects comparable to inhaled CO and as small molecules are much less cumbersome for administering and have the potential for targeted delivery, with different formulations exhibiting varying release kinetics [112,149]. Beyond metal carbonyls and organic compounds, gas-entrapping materials (GEMs) that encapsulate CO within GRAS-classified foams, hydrogels, or solid matrices permit non-inhalational delivery. Recent work has noted CO-independent activities of certain CORMs, including catalase-like activity, thiol reactivity, and NAD(P)^+^ reduction, which complicate mechanistic interpretation and argue for the use of inactivated-CORM controls in key mechanistic studies [149,150,151]. Importantly, these findings do not invalidate the renoprotective effects reported for CORM-2, CORM-3 [152], CORM-A1 [153], and CORM-401 [154] in experimental models of renal IRI and transplantation, but rather raise questions about the extent to which these effects can be specifically attributed to CO release versus CO-independent activities of the compounds. A similar argument follows work with CO-Prodrugs that also show kidney protection where off-target effects of the byproduct may occur after CO release [155]. Moreover, CO-independent chemical reactivity observed under defined experimental conditions or in cultured cells does not necessarily establish that pharmacological effects observed in vivo are likewise CO-independent as detailed tissue distribution studies for CO have not been reported.

Unlike CORMs or Prodrugs, the GEM form factors that have been recently characterized in detail hold approximately 0.9 mg CO per gram, roughly twenty-five times more than gas-enriched lactated Ringer’s solution or HBI-002 [87,156] and can be given orally, rectally or topically [147,157]. And unlike HBI-002, are easily tunable by using a variety of formulations and require smaller volumes. GEM are not CO-releasing agents, but rather CO incorporated into what are described as microbubbles that are highly tunable depending on the materials used. Thus, GEM differ significantly from the small molecule approaches described above. In rodents and swine, 5 g/kg produces a carboxyhemoglobin peak immediately after administration that declines over two hours, mirroring the kinetics of inhaled CO while remaining below 20%, with highest tissue accumulation in liver, heart, spleen, lung and kidney, precisely in the organs of interest discussed here [157]. Pairing a foam with a hyaluronic acid hydrogel yields biphasic release with an initial burst followed by sustained delivery over approximately two days [147].

### 5.2. Clinical Experience with CO Administration

Translation of CO therapy into clinical testing has progressed over the past two decades. In a phase I study of sepsis-induced ARDS, inhaled CO at 100–200 ppm for 90 min daily for up to five days was well tolerated, with carboxyhemoglobin remaining below 10% [158]. A phase IIa study in idiopathic pulmonary fibrosis similarly demonstrated that low-dose inhaled CO was well tolerated, although no significant improvement in clinical or physiological endpoints was observed [159]. In human islet autotransplantation, exposure of isolated islets to CO-saturated medium during the isolation procedure was associated with improved islet viability [118]. More recent clinical development includes oral CO (HBI-002) in sickle cell disease [[156], NCT06144749] and PEGylated carboxyhemoglobin (PP-007) in acute ischemic stroke [[160], NCT04677777]. A phase II study of inhaled CO in kidney transplantation (NCT00531856) was initiated but subsequently withdrawn, and no clinical efficacy results have been reported.

### 5.3. Machine Perfusion as a Novel Platform

Machine perfusion, both hypothermic and normothermic, has changed organ preservation and offers a natural platform for HO-1-based interventions [161,162,163,164,165,166]. During perfusion, the graft can be pharmacologically conditioned, transduced, or treated with CO gas or small molecules, with real-time assessment of viability [149]. Machine perfusion converts the graft into an isolated compartment for a defined interval between procurement and implantation, permitting testing of concentrations that would be unacceptable systemically while sparing the recipient entirely, and simultaneously supplying perfusate biochemistry as an interpretable intermediate endpoint. It also fits the operative timeline without adding a step, since the interval it occupies is otherwise dead time [167]. A CO-GEM offers a complementary form factor at this point, since a foam or hydrogel can be applied to the graft surface or introduced into the perfusate without the compressed-gas handling that inhalational delivery requires [147]. The principle has already been demonstrated in the extracorporeal setting where in a porcine model of cardiac arrest, CO was delivered directly into the extracorporeal resuscitation [168], establishing that a gas can be introduced into a running circuit and reach a target organ without exposing the airway. Machine perfusion has replaced static cold storage as the standard for marginal grafts, reducing delayed graft function and improving early outcomes in deceased donor kidney transplantation and sequential protocols that combine oxygenated hypothermic, controlled rewarming and normothermic perfusion in a single circuit now allow even high-risk donor livers to be resuscitated and assessed before implantation. Since the graft is already instrumented, monitored and separated from the recipient CO can be easily introduced in a safe and reliable manner.

## 6. Conclusions

The heme oxygenase-1 system imparts a critical endogenous defense against ischemia-reperfusion injury in organ transplantation. Through carbon monoxide, biliverdin and iron sequestration, HO-1 exerts multifaceted cytoprotective effects encompassing antioxidant defense, anti-inflammatory activity, inhibition of regulated cell death and maintenance of the microcirculation. Evidence from kidney, liver, lung, islet, small bowel, heart allotransplantation as well as xenotransplantation models consistently demonstrate that enhancing HO-1 expression or administering one or more of its products, particularly CO, improves short and long-term graft survival and function.

Kidney transplantation is the most compelling target primarily because delayed graft function is frequent and carries recognized consequences for long-term survival. Hypothermic and normothermic perfusion are already in clinical use, and the evidence spans rodent and swine allografts, large-animal ex vivo conditioning and the mechanistic convergence of heme, ferroptosis and tubular injury. Several questions remain open across the field and include: (1). whether recipient-derived or graft-derived HO-1 governs long-term alloimmunity. (2). what determines whether HO-1 activity suppresses or promotes ferroptosis and (3). how sustained HO-1 induction interacts with the elevated malignancy risk of chronically immunosuppressed recipients. The enormous volume of preclinical evidence showing the benefits of HO-1/CO in preserving graft function can now drive focused clinical trials. Some challenges that remain include: (1). donor treatment logistics that depend on the location of the organ to be harvested, (2). treatment of a donor means all organs will be exposed, (3). dose and dose regimen, (4). whether the recipient requires treatment to modulate the immune response and (5). the optimal modality to dose. Current data argue strongly for a GEM given the tunability, simplicity, delivery route options and even psychosocial aspects of CO as a therapy. This last point is becoming less and less of a concern given the rigorous safety studies that have been reported in both healthy and sick subjects showing no adverse events. Collectively, the time for HO-1/CO to become a standard of care for organ transplantation has arrived and will increase the number of available organs and may even reduce the amount of immunosuppression required.

Amidst all this positive and encouraging data, several challenges have constrained the clinical use of CO therapeutics, including concerns surrounding systemic carboxyhemoglobin levels, reported effects on the brain and heart, the narrow margin between the anti-inflammatory and the hypoxemic dose, the regulatory and perceptual burden of those administering CO to a human subject as well as the perception of CO as something to avoid. All of these concerns have been systematically argued away with novel alternatives to inhaled gas (detailed above), clear toxicology, pharmacology, as well as rigorous safety trials. In fact, the effects of CO on the brain and heart have not been reproduced in any of the Phase trials to date. None of the expected neurocognitive or cardiac events were reported up to carboxyhemoglobin (COHb) levels of 12–14% where most literature reports effects on cardiac perfusion, dizziness, nausea, and even altered mental status. This raises the question, if not concern, as to how best to interpret the abundant historical literature describing the deleterious effects of CO at even 2–3% COHb.

Taken together, HO-1 and its enzymatic products, particularly carbon monoxide, are clearly central regulators of graft survival, capable of limiting ischemia-reperfusion injury, curbing innate and adaptive alloimmune responses, and extending both early and long-term outcomes across multiple solid organ transplant models. As strategies to harness this pathway move from bench to bedside, through donor preconditioning, machine perfusion, and CO-based therapeutics, the HO-1 system continues to stand poised to translate decades of mechanistic insight into tangible improvements in transplant recipient care.

## Figures and Tables

**Table 1:** Hemoproteins contributing to the labile heme pool.

Hemoprotein	Category	Heme Content	Primary Function	Contribution to Pool	Location	Ref.
Hemoglobin	O_2_ transport and storage	Heme b (×4)	Oxygen transport in red blood cells	Dominant source; approximately 6–8 g of hemoglobin releases ~300 mg of heme per day; major contributor to RBC senescence after 120-day lifespan	Erythrocytes	[44]
Myoglobin	O_2_ transport and storage	Heme b	Oxygen storage and release in muscle	Significant source, especially in rhabdomyolysis; released upon acute muscle injury	Skeletal and cardiac muscle	[44,49]
Neuroglobin	O_2_ transport and storage	Heme b	O_2_ supply to neurons; neuroprotection; NO scavenging	Minor; localized to CNS neurons and endocrine cells	Brain neurons	[50]
Cytoglobin	O_2_ transport and storage	Heme b	O_2_ storage in fibroblasts; ROS scavenging; tumor suppression	Localized; released under oxidative stress	Fibroblasts; liver stellate cells	[50,51]
Cytochrome c	Electron transfer	Heme c	Electron shuttle in mitochondrial ETC (complex III → IV); initiates apoptosis on release	Released to cytosol during apoptosis, contributing to labile heme pool	Mitochondrial intermembrane space	[44,49]
Cytochrome b (complex III)	Electron transfer	Heme b (×2)	Core of bc1 complex; proton pumping and electron transfer	Membrane-bound; contributes on mitochondrial turnover/autophagy	Inner mitochondrial membrane	[44]
Cytochrome c1 (complex III)	Electron transfer	Heme c1	Electron transfer within bc1 complex to cytochrome c	Minor; liberated on mitochondrial degradation	Inner mitochondrial membrane	[44]
Cytochrome a/a3 (complex IV)	Electron transfer	Heme a (×2)	Terminal electron acceptor; reduces O_2_ to H_2_O; proton pumping	Minor; released on mitochondrial turnover	Inner mitochondrial membrane	[44]
Cytochrome b5	Electron transfer	Heme b5	Electron donor to desaturases, CYP450s and methemoglobin reductase	ER resident; contributes to microsomal turnover	ER membrane and outer mitochondria	[52]
Succinate dehydrogenase (complex II)	Electron transfer	Heme b	TCA cycle enzyme and ETC component; oxidizes succinate; generates ROS in ischemia	Contributes to mitochondrial autophagy; implicated in ROS during ischemia–reperfusion	Inner mitochondrial membrane	[44]
Cytochrome P450 family (57 human isoforms)	Enzymatic	Heme b	Drug/xenobiotic metabolism; steroid and fatty acid hydroxylation (e.g., CYP3A4, CYP2D6, and CYP1A2)	Major hepatic heme sink; >50% of hepatic heme is committed to P450 synthesis; significant pool contributor on CYP induction or degradation by drugs/toxins	ER and mitochondria; predominantly liver	[44,52]
Catalase	Enzymatic	Heme b (×4)	Decomposition of H_2_O_2_ to H_2_O + O_2_; antioxidant defense	Peroxisomal pool; contributes to protein turnover	Peroxisomes; all tissues	[44,49]
Myeloperoxidase (MPO)	Enzymatic	Heme b (modified)	Generates hypochlorous acid for microbial killing in neutrophils	Released in neutrophil degranulation or cell death; local inflammatory contribution	Neutrophil azurophilic granules	[44,49]
Eosinophil peroxidase (EPX)	Enzymatic	Heme b	Produces reactive oxidants in eosinophil-mediated immunity	Minor; localized to eosinophils; released during allergic inflammation	Eosinophil granules	[49]
Lactoperoxidase	Enzymatic	Heme b	Antimicrobial oxidant generation in secretions	Minor systemic contribution	Saliva, milk, and tears	[49]
Thyroid peroxidase (TPO)	Enzymatic	Heme b	Iodination and coupling of thyroid hormone precursors	Localized to thyroid; minor systemic contribution	Thyroid follicular cells	[49]
Tryptophan 2,3-dioxygenase (TDO)	Enzymatic	Heme b	First step in tryptophan catabolism (kynurenine pathway); regulates heme synthesis via feedback on ALAS1	Major hepatic heme-consuming enzyme; short half-life of the order of 2 h; holoenzyme stabilized by its heme cofactor and, therefore, highly sensitive to heme pool levels	Liver cytosol	[49,53]
Indoleamine 2,3-dioxygenase (IDO)	Enzymatic	Heme b	Tryptophan catabolism in immune/inflammatory contexts; immunosuppression via kynurenine pathway	Moderate; induced in inflammation; competes with TDO for heme at sites of infection	Immune cells, gut, and brain	[49,54]
Cystathionine beta-synthase (CBS)	Enzymatic	Heme b (regulatory)	H_2_S generation; trans-sulfuration pathway; homocysteine-to-cystathionine conversion	Regulatory heme binding; low quantitative contribution to labile pool	Liver, brain, and kidney	[44]
Cyclooxygenases 1 and 2 (COX-1/COX-2)	Enzymatic	Heme b	Prostaglandin synthesis from arachidonic acid; inflammation and hemostasis	Minor; localized; COX-2 induction in inflammation may modestly increase local heme demand	ER; platelets (COX-1) and induced tissues (COX-2)	[44,55]
Nitric oxide synthase (eNOS, nNOS, and iNOS)	Signaling	Heme b (×1 per monomer)	NO synthesis from L-arginine; vasodilation, neurotransmission, and immune defense	Minor in absolute terms; heme essential for dimerization and activity; and iNOS induction increases demand	Endothelium (eNOS), neurons (nNOS), and immune cells (iNOS)	[44]
Soluble guanylate cyclase (sGC)	Signaling	Heme b	NO sensor; converts GTP to cGMP on NO binding; and vasodilation signal transduction	Minor; heme essential for NO sensing; and heme loss renders enzyme NO-insensitive	Vascular smooth muscle; platelets	[44]
Heme-regulated inhibitor kinase (HRI)	Regulatory	Heme b (×2)	eIF2α kinase; heme sensor coordinating globin translation with heme availability in erythroid cells	Prevents globin chain synthesis when heme is low	Erythroid precursors	[49,56]
NPAS2/mPER2/Rev-Erbβ	Regulatory	Heme b	Heme-dependent transcription factors regulating circadian rhythm; CO/NO gas sensors	Minor quantitative role; heme acts as regulatory ligand altering gene transcription timing	Suprachiasmatic nucleus; liver	[57]
BACH1	Regulatory	Heme b	Heme-regulated repressor of HO-1, globin and ALAS; dissociates from DNA when heme levels are high	Critical feedback regulator linking heme abundance to HO-1 induction	Nuclear	[58,59]

**Table 2:** HO-1 and carbon monoxide cytoprotective mechanisms across transplanted organs.

Organ	Primary IRI Mechanism	How HO-1/CO Counteracts IRI	Key Mediators	Functional Outcome	Ref.
Kidney	Tubular necrosis; ATP depletion → tubular cell death; free heme release; inflammatory cell infiltration	CO causes vasodilation → restores renal blood flow CO inhibits apoptosis via p38 MAPK pathway	CO (vasodilation, anti-apoptosis)	↑ * GFR ↑ Perfusion flow ↓ * Tubular apoptosis ↓ Chronic nephropathy	[76,77,83,84,103,104,105,106]
Liver	Kupffer cell activation; ROS/cytokine release; sinusoidal congestion; hepatocyte apoptosis	Degrades pro-oxidant free heme CO suppresses TNF-α and IL-6 via p38 MAPK Biliverdin/bilirubin scavenge ROS Ferritin sequesters labile iron	CO, biliverdin, bilirubin, ferritin	↓ Apoptosis ↓ Oxidative stress ↓ Sinusoidal injury ↑ Graft survival	[78,79,94,107,108]
Heart	Cardiomyocyte death; Ca^2+^ overload; platelet aggregation; endothelial dysfunction; microvascular thrombosis	CO inhibits platelet aggregation via guanylate cyclase/cGMP CO suppresses endothelial apoptosis CO improves microcirculation by vasodilation	CO (cGMP)	↓ Platelet aggregation ↓ Graft arteriosclerosis ↑ Cardiac allograft survival	[4,81,86,109,110,111]
Lung	Alveolar-capillary damage; neutrophil infiltration; oxidative burst; fibrin deposition; pulmonary edema	CO markedly reduces neutrophil influx into airways CO attenuates apoptotic index in lung parenchyma p38 MAPK-dependent anti-inflammatory effects Prevents fibrin deposition and pulmonary edema	CO (p38 MAPK, anti-apoptosis, anti-neutrophil)	↓ Neutrophil influx ↓ Fibrin deposition ↓ Pulmonary edema ↑ Graft function	[93,112,113,114,115,116]
Islets	β-cell apoptosis; inflammatory cytokines (IL-1β, TNF-α) trigger β-cell death; oxidative damage post-transplant	HO-1 overexpression or CO protects β-cells CO suppresses IL-1β/TNF-α production via MAPK Bilirubin scavenges cytokine-induced ROS CO inhibits β-cell apoptosis, preserving insulin secretion	CO, bilirubin	↓ β-cell apoptosis ↑ Islet engraftment ↑ Glycemic control	[117,118,119]
Small bowel	Mucosal barrier failure; epithelial apoptosis; villus atrophy; oxidative stress; leukocyte recruitment in graft microvasculature	HO-1 induction or CO protects intestinal epithelium CO reduces epithelial apoptosis CO/HO-1 suppress inflammatory cytokines, preserving mucosal barrier	CO, biliverdin	↓ Epithelial apoptosis ↑ Mucosal integrity ↓ Leukocyte recruitment ↑ Graft survival	[120,121]
Skin	T-cell-mediated rejection; leukocyte rolling and adhesion; ICAM-1 upregulation; endothelial permeability; DC-driven immune activation	HO-1 reduces leukocyte adhesion to endothelium CO causes arteriole dilation and restores capillary perfusion HO-1 inhibits ICAM-1 expression and endothelial permeability CO suppresses dendritic cell maturation → immune tolerance	CO (vasodilation), biliverdin (anti-inflammatory)	↓ Leukocyte adhesion ↓ ICAM-1 ↑ Capillary perfusion ↑ Allograft survival	[122]

* ↑ indicates increased expression, activity, or effect; ↓ indicates decreased expression, activity, or effect.

## Data Availability

No new data were created or analyzed in this study. Data sharing is not applicable to this article.

## Abbreviations

The following abbreviations are used in this manuscript:

ALAS1	5-aminolevulinate synthase 1
ARDS	Acute respiratory distress syndrome
ATP	Adenosine triphosphate
BACH1	BTB and CNC homology 1
CBS	Cystathionine beta-synthase
cGMP	Cyclic guanosine monophosphate
CNS	Central nervous system
CO	Carbon monoxide
COHb	Carboxyhemoglobin
CO-RM	Carbon monoxide-releasing molecule
COX	Cyclooxygenase
CYP	Cytochrome P450
DAMP	Damage-associated molecular pattern
DC	Dendritic cell
eIF2α	Eukaryotic initiation factor 2α
eNOS	Endothelial nitric oxide synthase
EPX	Eosinophil peroxidase
ER	Endoplasmic reticulum
ETC	Electron transport chain
GEM	Gas-entrapping material
GFR	Glomerular filtration rate
HMOX1	Heme oxygenase-1 gene
HO-1	Heme oxygenase-1
HO-2	Heme oxygenase-2
HRI	Heme-regulated inhibitor kinase
ICAM-1	Intercellular adhesion molecule-1
IDO	Indoleamine 2,3-dioxygenase
IL	Interleukin
iNOS	Inducible nitric oxide synthase
IRI	Ischemia-reperfusion injury
KEAP1	Kelch-like ECH-associated protein 1
MAPK	Mitogen-activated protein kinase
MD-2	Myeloid differentiation factor 2
MLKL	Mixed lineage kinase domain-like protein
MPO	Myeloperoxidase
mPTP	Mitochondrial permeability transition pore
MyD88	Myeloid differentiation primary response 88
NF-κB	Nuclear factor-κB
nNOS	Neuronal nitric oxide synthase
NO	Nitric oxide
NRF2	Nuclear factor erythroid 2-related factor 2
ppm	Parts per million
RBC	Red blood cell
RIPK	Receptor-interacting protein kinase
ROS	Reactive oxygen species
sGC	Soluble guanylate cyclase
TCA	Tricarboxylic acid
TDO	Tryptophan 2,3-dioxygenase
TGF-β	Transforming growth factor-β
TLR4	Toll-like receptor 4
TNF	Tumor necrosis factor
TPO	Thyroid peroxidase
UW	University of Wisconsin
VCAM-1	Vascular cell adhesion molecule-1
